# Pretreatment systemic inflammation response index is predictive of pathological complete response in patients with breast cancer receiving neoadjuvant chemotherapy

**DOI:** 10.1186/s12885-021-08458-4

**Published:** 2021-06-14

**Authors:** Jie Dong, Qingqing Sun, Yueyin Pan, Nannan Lu, Xinghua Han, Qiong Zhou

**Affiliations:** 1 Department of Medical Oncology, Anhui Provincial Hospital affiliated to Anhui Medical University, Hefei, 2300001 Anhui Province China; 2 Department of Medical Oncology, The First Affiliated Hospital of USTC, Division of Life Sciences and Medicine, University of Science and Technology of China, Hefei, 230001 Anhui Province China

**Keywords:** Breast cancer, Neoadjuvant chemotherapy, Systemic inflammation response index, SIRI, Pathological complete response

## Abstract

**Background:**

Inflammation plays an important role in tumor proliferation, metastasis, and resistance to chemotherapy. The systemic inflammation response index (SIRI), has been reported to be closely related to prognosis in many tumors, such as breast and gastric cancers. However, the predictive value of pretreatment SIRI on pathological complete response (pCR) rates in patients with breast cancer treated with neoadjuvant chemotherapy (NAC) is unknown. This study examined the correlation between SIRI and pCR in patients with breast cancer receiving NAC and identified convenient and accurate predictive indicators for pCR.

**Methods:**

We retrospectively analyzed the clinicopathological parameters and pretreatment peripheral blood characteristics of the 241 patients with breast cancer who received NAC between June 2015 and June 2020. Receiver operating characteristic (ROC) curves were used to determine the optimal cutoff of SIRI. ROC curves were also plotted to verify the accuracy of inflammatory markers for pCR prediction. The chi-squared test was used to explore the relationships of SIRI with pCR and other clinicopathological parameters. Multivariate analyses were performed using a logistic regression model.

**Results:**

Among the 241 patients, 48 (19.92%) achieved pCR. pCR was significantly related to SIRI, the neutrophil-lymphocyte ratio (NLR), the lymphocyte-monocyte ratio (LMR), molecular subtypes and other clinicopathological parameters, such as BMI, clinical T and N staging, and histological grade. Multivariate analyses indicated that the clinical T and N staging, SIRI, and NLR were independent prognostic factors for pCR in patients with breast cancer. The area under the ROC curve for SIRI was larger than that for NLR. Compared to patients with SIRI ≥0.72, patients with SIRI < 0.72 had a nearly 5-fold higher chance of obtaining pCR (odds ratio = 4.999, 95% confidence interval = 1.510–16.551, *p* = 0.000).

**Conclusions:**

Pretreatment SIRI is predictive of pCR in patients with breast cancer receiving NAC, and the index can assist physicians in formulating personalized treatment strategies.

**Supplementary Information:**

The online version contains supplementary material available at 10.1186/s12885-021-08458-4.

## Background

Breast cancer is the most commonly diagnosed malignant tumor and leading major cause of cancer-related death in women [1]. Neoadjuvant chemotherapy (NAC), which was introduced toward the end of the twentieth century [2], aims to downstage locally advanced disease and make it operable, and it was subsequently extended to early breast cancer, in which it significantly improves the breast-conserving surgery rate [3, 4].

Pathological complete response (pCR) is widely used as a surrogate endpoint in patients with breast cancer who received NAC with the aim of accelerating the evaluation of new treatment strategies, which is closely related to disease-free survival (DFS) and overall survival (OS) [5, 6]. A pooled analysis of the collaborative neoadjuvant breast cancer trial (CTNeoBC) illustrated that patients with pCR had a significantly longer survival time than those with residual tumors [7]. The response to NAC is critical for predicting prognosis and choosing the optimal chemotherapies to avoid excessive therapy. Hence, it is of great importance to predict pCR in patients with breast cancer receiving NAC.

Inflammation, recognized as one of the 10 hallmarks of cancer, contributes to tumor proliferation, angiogenesis, metastasis, and resistance to chemotherapy. Several studies revealed that systemic inflammation markers, such as the neutrophil-lymphocyte ratio (NLR) and lymphocyte-monocyte ratio (LMR), are associated with pCR in breast cancer [8, 9]. The systemic inflammation response index (SIRI) is an integrated indicator based on the counts of peripheral venous blood neutrophils, monocytes and lymphocytes that might reflect the status of the local immune response and systemic inflammation [10]. To date, the relationship between SIRI and pCR has rarely been reported [11]. SIRI was first reported by Qi et al. for its ability to predict the survival of patients with pancreatic cancer [10]. Later, SIRI was confirmed to be related to prognosis in many tumors such as gastric and breast cancer [12, 13]. However, the predictive value of SIRI for pCR remains unclear in breast cancer.

In view of these findings, this study is examined whether SIRI is a predictive factor for pCR in patients with breast cancer.

## Methods

### Patients

We retrospectively enrolled 241 patients with primary breast cancer who received NAC at the First Affiliated Hospital of USTC between June 2015 and June 2020. We extracted detailed treatment information and clinical data for all patients from their medical records. The project was approved by the ethics committee of The First Affiliated Hospital of USTC. The requirement for informed consent was waived because of the retrospective nature of the study.

The inclusion criteria were as follows: (1) the availability of complete blood test results and pathological information; (2) receipt of NAC after diagnosis and surgical treatment, such as breast-conserving surgery or modified radical mastectomy; and (3) diagnosis via needle core biopsy before NAC.

**Fig. 1:**
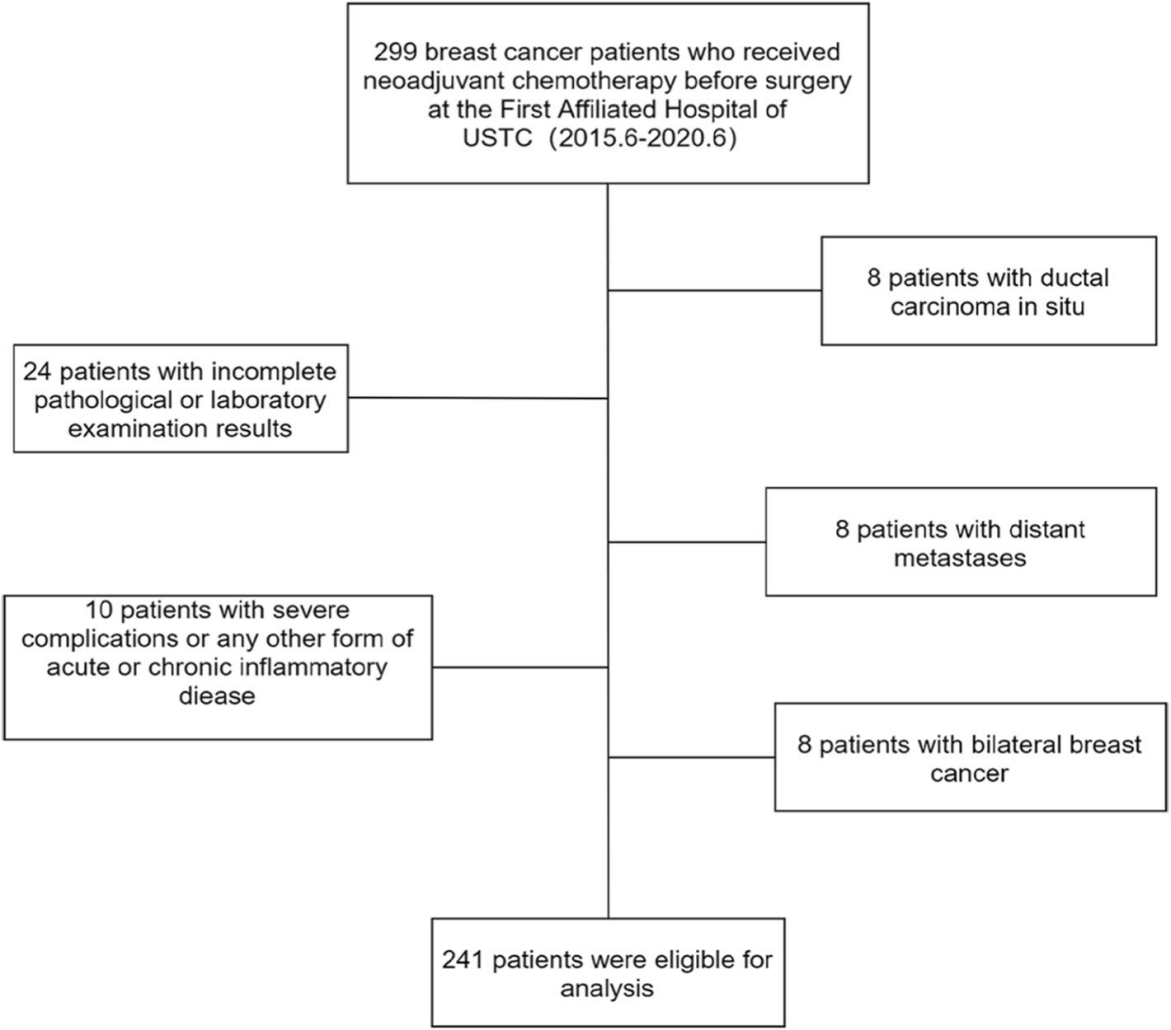
241 breast cancer patients were collected and the exclusion criteria

The exclusion criteria were as follows: (1) ductal carcinoma in situ irrespective of the presence of microinvasion; (2) incomplete pathological or laboratory examination results; (3) distant metastases; (4) bilateral breast cancer or inflammatory breast cancer; (5) severe inflammatory diseases or complications. This study included patients receiving neoadjuvant therapy with sequential regimens featuring anthracyclines and/or taxanes. Patients with human epidermal growth factor receptor 2 (HER-2) overexpression received anti-HER-2 targeted neoadjuvant therapy (Fig. 1).

### Blood samples

In all patients, peripheral venous blood samples were collected on an empty stomach within 1 week before NAC. SIRI is calculated using the counts of peripheral venous blood neutrophils (N), monocytes (M), and lymphocytes (L) as follows: SIRI=N × M/L. NLR, LMR and the absolute lymphocyte count (ALC) were determined. We performed all peripheral venous blood cells evaluations in our local institutional laboratory.

### Pathology

We used the 8th edition of the American Joint Committee on Cancer staging manual to determine the clinical staging of all patients in this study. The estrogen receptor (ER) and progesterone receptor (PR) status were assessed via immunohistochemistry, and positivity was indicated by the staining of at least 1% of tumor cell nuclei in the sample. The HER-2 status was assessed via immunohistochemistry and/or fluorescence in situ hybridization. This study describes the criteria for the definition of HER-2 positivity [14]. According to the results of ER, PR, and HER-2 immunohistochemical staining, the molecular subtypes were divided into three groups: triple-negative (hormone receptor [HR]−/HER-2−), HER-2–positive (HR±/Her-2+) and HR–positive (HR+/HER-2–). We defined pCR as the absence of residual invasive cancer in surgical pathologic specimens (ypT0/ypTis and ypN0) [15].

### Statistical analysis

Receiver operating characteristic (ROC) curve analysis was used to determine the optimal cutoffs for inflammatory markers (SIRI, NLR, LMR, ALC), and the ratio of the point with the highest Youden’s index was defined as the best cutoff. ROC curves were also plotted to verify the accuracy of inflammatory markers for pCR prediction. The chi-squared test was used to assess the relationship between clinicopathological parameters and pCR. Logistic regression analysis was performed for multivariate analysis using an enter method. Statistical analysis was performed using SPSS version 22.0 software (IBM Corporation, Armonk, NY, USA) and GraphPad Prism version 7.0 software (GraphPad Software Inc., San Diego, CA, USA). *P* < 0.05 was considered statistically significant.

## Results

### Clinicopathological parameters of all patients

**Fig. 2:**
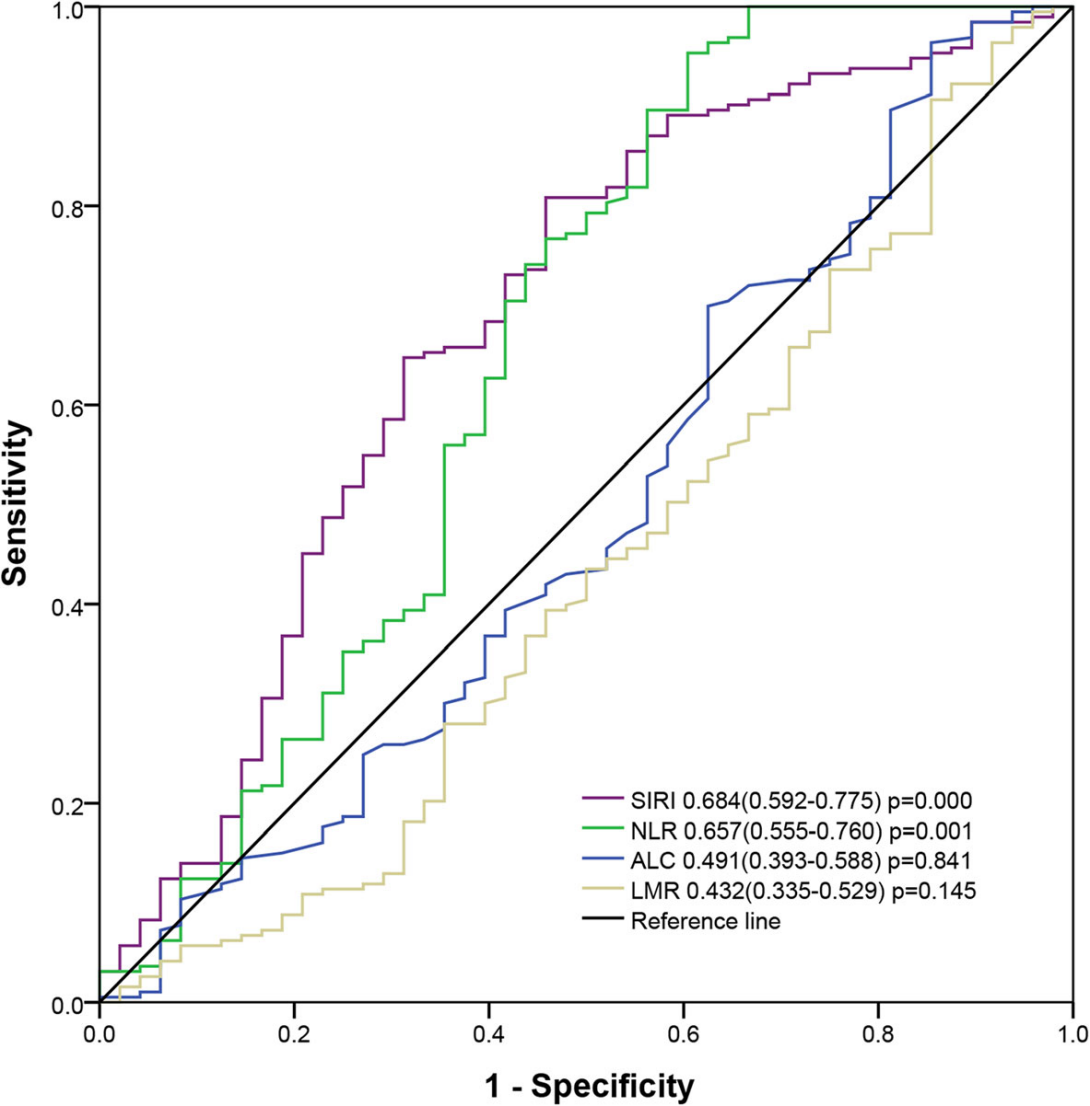
Predictive ability of the SIRI was compared with NLR, ALC and LMR by ROC curves

We identified 299 patients with breast cancer who received NAC. According to the inclusion and exclusion criteria, 241 patients were finally included. The optimal cutoff of SIRI was 0.72 × 10^9^/L (area under the ROC curve [AUC] = 0.684,,95% confidence interval [CI] = 0.592–0.775, *p* = 0.000) (Fig. 2). The median age at diagnosis was 48 years (range, 23–74). The low and high SIRI groups included 63 (26.14%) and 178 patients (73.86%), respectively. Most patients were diagnosed with cT2 lesions on the basis of tumor size (68.88%), and 65.15% of patients had cN1-N3 lesions. Only 1.24% of tumors were well-differentiated (G1). Triple-negative breast cancer was detected in 53 patients (22.00%). HER-2–positive was identified in 87 patients (36.10%), and HR–positive was identified in 101 patients (41.90%). Forty-eight patients (19.92%) achieved pCR after NAC. The detailed clinicopathological parameters of all patients are presented in Table 1.

**Table 1:** Patients’ clinicopathological parameters (*n* = 241)

Variables	Number	Percent (%)
**Age (years)**		
< 48	108	44.81
≥ 48	133	55.19
**Body mass index (kg/m2)**		
< 24	125	51.90
≥ 24	116	48.10
**Clinical T stage**		
T1	26	10.79
T2	166	68.88
T3-T4	49	20.33
**Clinical N stage**		
N0	84	34.85
N1–N3	157	65.15
**Grade**		
Grade1	3	1.24
Grade2	120	49.79
Grade3	70	29.05
UN	48	19.92
**Phenotype by IHC**		
HR-positive	101	41.90
HER-2-positive	87	36.10
Triple-negative	53	22.00
**Ki67 proliferation index**		
≤ 20	69	28.63
> 20	172	71.37
**Nac regimens**		
Anthra based	39	16.18
Tax based	25	10.37
Anthra + Tax based	177	73.44
**SIRI**		
< 0.72	63	26.14
≥ 0.72	178	73.86
**NLR**		
< 1.77	28	11.62
≥ 1.77	213	88.38
**LMR**		
< 5.38	201	83.40
≥ 5.38	40	16.60
**ALC**		
< 1.57	132	54.77
≥ 1.57	109	45.23
**Response to NAC**		
pCR	48	19.92
N-pCR	193	80.08

### Relationship between clinicopathological parameters and pCR

After NAC, 48 patients achieved pCR. In the present study, several factors were related to pCR, such as BMI, clinical T and N staging, histological grade, molecular subtypes, SIRI, NLR and LMR (Table 2). Interestingly, patients with low BMI had a significantly higher pCR rate than those with high BMI. In addition, higher pCR rates were observed in patients with higher clinical T and N stages. Patients with triple-negative breast cancer and HER-2–positive breast cancer had higher pCR rates than those with HR-positive breast cancer. Furthermore, the pCR rate was higher in patients with low SIRI than in those with high SIRI. Consistently, patients with low NLR had a higher pCR rate. Additionally, the pCR rate was also higher in patients with high LMR. Multivariate analysis illustrated that only clinical T and N staging, NLR, and SIRI retained their importance (Table 3). The AUC for SIRI was larger than that for NLR (Fig. 2). Patients with SIRI < 0.72 had an nearly 5-fold higher chance of achieving pCR than those with SIRI ≥0.72 (odds ratio = 4.999, 95% CI = 1.510–16.551, *p* = 0.000).

**Table 2:** Associations of clinicopathological parameters with pCR in breast cancer patients

Variables	N-pCR	pCR (N, %)	***P***-value
**Age (years)**			
< 48	84	24 (22.22)	0.419
≥ 48	109	24 (18.05)	
**Body mass index (kg/m2)**			
< 24	94	31 (24.80)	0.049
≥ 24	99	17 (14.66)	
**Clinical T stage**			
T1	16	10 (38.46)	0.043
T2	137	29 (17.47)	
T3-T4	40	9 (18.37)	
**Clinical N stage**			
N0	47	37 (44.05)	0.000
N1–N3	146	11 (7.01)	
**Grade**			
Grade1	2	1 (33.33)	0.013
Grade2	104	16 (13.33)	
Grade3	56	14 (20.00)	
UN	31	17 (35.41)	
**Phenotype by IHC**			
HR-positive	85	16 (15.84)	0.042
HER-2-positive	72	15 (17.24)	
Triple-negative	36	17 (32.07)	
**Ki67 proliferation index**			
≤ 20	59	10 (14.49)	0.182
> 20	134	38 (22.09)	
**Nac regimens**			
Anthra based	29	10 (25.64)	0.291
Tax based	18	7 (28.00)	
Anthra + Tax based	146	31 (17.51)	
**SIRI**			
< 0.72	37	26 (41.27)	0.000
≥ 0.72	156	22 (12.36)	
**NLR**			
< 1.77	9	19 (67.86)	0.000
≥ 1.77	184	29 (13.62)	
**LMR**			
< 5.38	168	33 (16.41)	0.002
≥ 5.38	25	15 (37.50)	
**ALC**			
< 1.57	109	23 (21.10)	0.286
≥ 1.57	84	25 (22.94)

**Table 3:** Predictive factors for pCR in multivariate analyses

Variables	Multivariate OR (95% CI)	***P***-value
**Body mass index (kg/m2)**		
< 24	1	
≥ 24	0.577 (0.232–1.437)	0.238
**Clinical T stage**		
T1	1	
T2	0.276 (0.082–0.927)	0.037
T3-T4	0.221 (0.053–0.927)	0.039
**Clinical N stage**		
N0	1	
N1-N3	0.063 (0.022–0.176)	0.000
**Grade**		
Grade1	1	
Grade2	0.499 (0.023–10.820)	0.658
Grade3	0.698 (0.030–15.977)	0.822
U N	2.186 (0.093–51.421)	0.628
**Phenotype by IHC**		
HR-positive	1	
HER-2-positive	0.658 (0.220–1.969)	0.454
Triple-negative	2.110 (0.697–6.385)	0.186
**SIRI**		
≥ 0.72	1	
< 0.72	4.999 (1.510–16.551)	0.008
**NLR**		
≥ 1.77	1	
< 1.77	7.257 (2.122–24.818)	0.002
**LMR**		
< 5.38	1	
≥ 5.38	0.616 (0.181–2.090)	0.437

## Discussion

Cancer-related inflammation is of great importance to the occurrence and development of tumors and resistance to chemotherapy [16]. As an indicator of inflammation, SIRI has been reported to be closely related to the prognosis in breast cancer. However, the correlation between SIRI and pCR in patients with breast cancer remains unclear. This study described the predictive value of SIRI for pCR.

In the present study, parameters such as SIRI, NLR, LMR, BMI, clinical T and N staging, histological grade, and molecular subtypes were significantly associated with pCR. However, multivariate analysis illustrated that only SIRI, NLR, and clinical T and N staging were independently predictive of pCR among those parameters. Inflammation cells have important anti-tumor functions. Neutrophils (both circulating and tumor-associated) can acquire an anti-tumor phenotype in the presence of interferon-β or upon the inhibition of transforming growth factor-β signaling [17, 18]. Lymphocytes can suppress tumor growth through direct cytotoxicity, apoptosis induction, and angiogenesis inhibition [19]. Monocytes exert anti-tumor effects through phagocytosis, tumoricidal mediator secretion, lymphocyte recruitment, and differentiation into tumor-associated macrophages and dendritic cells [20]. Systemic inflammation can affect a patient’s response to chemotherapeutic agents [21]. NLR has been introduced as a predictive factor for pCR in patients with breast cancer who receive NAC [22]. SIRI was reported to be related to prognosis in many cancer types such as nasopharyngeal cancer and pancreatic cancer [23, 24]. Consistent with previous reports, our study also found that SIRI can indicate the prognosis of breast cancer. This is also the first study reporting that SIRI is predictive of pCR. SIRI has been reported to reflect the status of the local immune response and systemic inflammation [10]. Compared to NLR, SIRI is more comprehensive because monocytes were also included in the model. In addition, the interactions among the three inflammatory cell types might enhance the prognostic value of the indicator. In the present study, the AUC for SIRI was larger than that for NLR, indicating that the prognostic value of the SIRI for pCR was superior to that of NLR. Although some detection methods, such as NLR, MRI, PET/CT, have been reported to predict pCR, in actual clinical practice no detection method has been used to predict pCR [25, 26]. Hence, we must keep exploring new indicators for clinical practice. Compared to NLR, SIRI is a new comprehensive indicator for pCR with greater potential value for clinical practice. In summary, SIRI has more advantages than NLR.

Some studies and clinical trials illustrated that imaging, positron emission tomography, and gene expression profiling such as PIK3CA mutation can predict the outcome of neoadjuvant chemotherapy [27–29]. However, these methods or indicators are costly and not conducive to general clinical implementation. SIRI, as an immune/inflammation index, is based on the counts of peripheral blood neutrophils, monocytes, and lymphocytes, and it has a simple and inexpensive procedure. In this study, we provided evidence that SIRI was independently predictive of pCR. By calculating SIRI, we can predict whether a patient will quickly achieve pCR, permitting the selection of an optimal treatment strategy for each patient. In addition, we can also save medical resources and reduce the financial burden of patients.

The pathophysiological functions of peripheral venous blood cells, such as neutrophils, monocytes and lymphocytes, may explain the mechanisms by which SIRI is predictive of pCR in patients with breast cancer receiving NAC. First, neutrophils produce substances, such as chemokines and/or cytokines, matrix-degrading proteases and reactive oxygen species, which can alter tumor growth and aggressiveness. Several studies indicated that neutrophils promote tumor progression through matrix degradation and cancer cell proliferation [16]. Second, monocytes, especially tumor-associated macrophages, are the mediators of the link between inflammation and cancer. They also play multiple roles in various stages of cancer development [20]. Third, lymphocytes can inhibit tumor cell proliferation and migration by inducing cytotoxic cell death, which plays a key role in tumor immune monitoring and defense [30]. For patients with cancer and fever exceeding 38 °C, night sweats, fatigue, weight loss of more than 5% (so-called B symptoms), and malnutrition, the clinical outcome is poor, and this outcome is related to increased circulating cytokine concentrations [31]. The levels of these factors reflect the host’s immune response to tumors. These reasons may explain why patients with higher SIRI have lower pCR rate.

Different studies reported that SIRI is closely related to prognosis in many tumors. Chen et al. found in nasopharyngeal carcinoma that patients with higher SIRI had significantly shorter OS than those with lower SIRI [24]. In addition, in postmenopausal women with breast cancer, Hua et al. reported that patients with low SIRI had significantly longer OS than those with high SIRI [32]. Consistently, Chen et al. observed that among patients with breast cancer treated with NAC, those with low SIRI had longer DFS and OS, but they did not find a statistical correlation between SIRI and pCR, which contradicts our results [11]. In patients with breast cancer, different ages, stages and phenotypes also correspond to different immune responses, therefore leading to different SIRIs. We believe that the differences in the characteristics of the enrolled population explain the differences between our results and previous findings. Therefore, multi-center prospective clinical studies are required to further confirm our results.

This study confirmed the predictive value of SIRI for pCR, but some limitations must be addressed. First, this was a relatively small, single-center, and retrospective study, and its conclusions may be biased. Second, there may have been deviations in the assessment of the inflammatory status. Various internal and external factors can affect SIRI, such as acute inflammation, chronic infection and lifestyle. More importantly, we failed to obtain follow-up data to assess the correlation of SIRI with DFS and OS. Therefore, large multi-center studies are needed for further confirmation.

## Conclusions

SIRI is a new comprehensive index based on the total numbers of peripheral neutrophils, monocytes, and lymphocytes; thus it can provide a non-invasive, easily accessible, reproducible, cost-effective and feasible method for predicting the response to NAC and assist physicians in formulating personalized treatment strategies.

## Supplementary Information

**Additional file 1: Supplementary Table 1.** Associations of clinicopathological features with SIRI in breast cancer.

## Data Availability

The datasets used and/or analyzed during the current study are available from the corresponding author on reasonable request.

## Abbreviations

ALC: The Absolute Lymphocyte Count
AUC: Area Under The ROC Curve
CI: Confidence Interval
L: Lymphocyte
LMR: The Lymphocyte-Monocyte Ratio
M: Monocyte
N: Neutrophil
NLR: The Neutrophil-Lymphocyte Ratio
NAC: Neoadjuvant Chemotherapy
pCR: pathological Complete Response
ROC: Receiver Operating Characteristic
SIRI: Systemic Inflammation Response Index
