# Supplementary material for: Pretreatment systemic inflammation response index is predictive of pathological complete response in patients with breast cancer receiving neoadjuvant chemotherapy
Source: BMC Cancer. 2021 Jun 14;21:700. doi: 10.1186/s12885-021-08458-4 (PMC8204500; doi:10.1186/s12885-021-08458-4)
Supplement: Supplementary file 1 — Additional file 1: Supplementary Table 1. Associations of clinicopathological features with SIRI in breast cancer. [file 12885_2021_8458_MOESM1_ESM.docx]

| **Supplementary Table 1 Associations of clinicopathological features with SIRI in**  **breast cancer.** | | | | | |  |
| --- | --- | --- | --- | --- | --- | --- |
| **Variables** | **SIRI≥0.72** | **SIRI<0.72** | **P-value** |  |  | |
|  |  |  |  |  |  | |
|  |  |  |  |  |  | |
| **Age (years)** |  |  |  |  |  | |
| <48 | 83 | 25 | 0.341 |  |  | |
| ≥48 | 95 | 38 |  |  |  | |
| **Body mass index (kg/m2)** | |  |  |  |  | |
| <24 | 88 | 37 | 0.205 |  |  | |
| ≥24 | 90 | 26 |  |  |  | |
| **Clinical T stage** |  |  |  |  |  | |
| T1 | 19 | 7 | 0.805 |  |  | |
| T2 | 121 | 45 |  |  |  | |
| T3-T4 | 38 | 11 |  |  |  | |
| **Clinical N stage** |  |  |  |  |  | |
| N0 | 57 | 27 | 0.121 |  |  | |
| N1–N3 | 121 | 36 |  |  |  | |
| **Grade** |  |  |  |  |  | |
| Grade1 | 2 | 1 | 0.418 |  |  | |
| Grade2 | 92 | 28 |  |  |  | |
| Grade3 | 53 | 17 |  |  |  | |
| UN | 31 | 17 |  |  |  | |
| **Phenotype by IHC** |  |  |  |  |  | |
| HR positive | 81 | 20 | 0.070 |  |  | |
| Her-2 positive | 57 | 30 |  |  |  | |
| TNBC | 40 | 13 |  |  |  | |
| **Ki67 proliferation index** | |  |  |  |  | |
| ≤20 | 50 | 19 | 0.755 |  |  | |
| >20 | 128 | 44 |  |  |  | |
| **Nac regimens** |  |  |  |  |  | |
| Anthra based | 29 | 10 | 0.974 |  |  | |
| Tax based | 18 | 7 |  |  |  | |
| Anthra + Tax based | 131 | 46 |  |  |  | |
|  |  |  |  |  |  | |
|  |  |  |  |  |  | |
|  |  |  |  |  |  | |
|  |  |  |  |  |  | |
